# Time-Dependent Size and Shape Evolution of Gold and Europium Nanoparticles from a Bioproducing Microorganism, a Cyanobacterium: A Digitally Supported High-Resolution Image Analysis

**DOI:** 10.3390/nano13010130

**Published:** 2022-12-27

**Authors:** Melanie Fritz, Susanne Körsten, Xiaochen Chen, Guifang Yang, Yuancai Lv, Minghua Liu, Stefan Wehner, Christian B. Fischer

**Affiliations:** 1 Department of Physics, University Koblenz-Landau, Universitätsstraße 1, D-56070 Koblenz, Germany; 2 Fujian Provincial Engineering Research Center of Rural Waste Recycling Technology, College of Environment & Resources, Fuzhou University, Fuzhou 350116, China; 3 Materials Science, Energy and Nano-Engineering Department, Mohammed VI Polytechnic University, Ben Guerir 43150, Morocco

**Keywords:** cyanobacteria, nanoparticle size distribution, digital image processing, growth monitoring, shape classification

## Abstract

Herein, the particle size distributions (PSDs) and shape analysis of in vivo bioproduced particles from aqueous Au^3+^ and Eu^3+^ solutions by the cyanobacterium *Anabaena* sp. are examined in detail at the nanoscale. Generally, biosynthesis is affected by numerous parameters. Therefore, it is challenging to find the key set points for generating tailored nanoparticles (NPs). PSDs and shape analysis of the Au and Eu-NPs were performed with *ImageJ* using high-resolution transmission electron microscopy (HR-TEM) images. As the HR-TEM image analysis reflects only a fraction of the detected NPs within the cells, additional PSDs of the complete cell were performed to determine the NP count and to evaluate the different accuracies. Furthermore, local PSDs were carried out at five randomly selected locations within a single cell to identify local hotspots or agglomerations. The PSDs show that particle size depends mainly on contact time, while the particle shape is hardly affected. The particles formed are distributed quite evenly within the cells. HR-PSDs for Au-NPs show an average equivalent circular diameter (ECD) of 8.4 nm (24 h) and 7.2 nm (51 h). In contrast, Eu-NPs preferably exhibit an average ECD of 10.6 nm (10 h) and 12.3 nm (244 h). Au-NPs are classified predominantly as “very round” with an average reciprocal aspect ratio (RAR) of ~0.9 and a Feret major axis ratio (FMR) of ~1.17. Eu-NPs mainly belong to the “rounded” class with a smaller RAR of ~0.6 and a FMR of ~1.3. These results show that an increase in contact time is not accompanied by an average particle growth for Au-NPs, but by a doubling of the particle number. *Anabaena* sp. is capable of biosorbing and bioreducing dissolved Au^3+^ and Eu^3+^ ions from aqueous solutions, generating nano-sized Au and Eu particles, respectively. Therefore, it is a low-cost, non-toxic and effective candidate for a rapid recovery of these sought-after metals via the bioproduction of NPs with defined sizes and shapes, providing a high potential for scale-up.

## 1. Introduction

Microbial in vitro or in vivo synthesized nanoparticles (NPs) are of great interest as they can be generated in an environmentally friendly and cost-effective manner. Especially in the field of biomedical applications, biogenic synthesis routes offer a sustainable and safe technique [1]. Bioproduction can be carried out under ambient temperatures, unlike conventional synthetic processes [2]. The microorganisms used as biological “nanofactories” are capable of accumulating gold (Au), rare earths (REs) and other dissolved elements from industrial waste or other anthropogenically contaminated sites. At the same time, they are able to satisfy the objectives of resource recovery and pollution reduction [3,4]. Eu ions accumulate in soil and water due to the gasoline industry, nuclear wastewater, or discarded equipment containing Eu dyes [5]. This harms the reproduction, nervous system and cell membranes of aquatic organisms and poses a potential threat to human health [6].

In particular, Au and RE recovery from mine waste is becoming increasingly attractive, as it is necessary to rehabilitate mine tailings using nature-based solutions and afterwards use them economically. The effort to discard the biomass contaminated with metals should be used profitably. For this purpose, the processing of plants to products is carried out, which is called farming for metals or agro-mining [7]. Rising mining costs also favor the biotechnological recycling of critical metals, which include Au and Eu. Biorecovery of Eu from primary (mineral deposits) and secondary (mining wastes) resources is of interest due to its scarcity and inherent luminescence properties [8]. Besides the recovery from mine tailings, industrial electronic waste, especially waste printed circuit boards [9], are attractive resources of trivalent actinides. E-waste, is one of the fastest increasing waste streams (>50 Mt in the year 2019, estimated 74 Mt in 2030), whose disposal and effective management is a global challenge according to the UN´s Global E-waste Monitor 2020 [10].

Some studies are conducted to find suitable “green” sorbents (e.g., cell walls of *Stephanopyxis turris* and *Thalassiosira pseudonana*) to remediate Eu and model sorption reaction by using diffuse double layer model [11]. In sorption, different uptake mechanisms are possible: surface adsorption and incorporation/precipitation. The extent of adsorption is known to depend on solution pH, contact time, metal ion concentration and adsorbent dose [12].

To the best of the authors’ knowledge, only few studies have been conducted on microbial Eu (nano-)particle synthesis concerning more the fact of demonstrating bioaccumulation and/or biosorption that can be used for biological recovery. Hence, Cadogan et al. successfully removed Eu^3+^ ions by *Arthrobacter* sp. by biomass and crab shell powder amongst others from aqueous solution through biosorption [5,12]. Serna et al. provided insights into the passive biosorption of Eu^3+^ binding properties to chemically distinct sites of different biomaterials using luminescence spectroscopy [13]. In contrast to the aforementioned methods, Kim et al. synthesized europium selenide (EuSe) NPs with recombinant *Escherichia coli* cells in vivo and showed the anti-cancer effect of these generated NPs [14]. Maleke et al. studied the reduction and intracellular bioaccumulation of Eu by a *Clostridium* strain and found it suitable for biorecovery of this critical metal [8].

In addition to intracellular uptake in vivo biosynthesis of NPs [15,16], there are methods in which stable crystalline Au particles are formed extracellularly in vitro using biomass extracts [17]. Commonly, in vitro methods for the quasi biosynthesis of (Au-)NPs by using biomass extracts are used to (extracellularly) generate particles.

The state of the art Au-NP biosynthesis is better established [18,19,20,21] than that for REs such as Eu. For example, Dahoumane et al. studied improvements of kinetics yield and colloidal stability of biogenic Au-NPs [21]. Furthermore, Castro et al. found that the initial pH value of the solution and the concentration of Au precursor influences the morphology and NP formation of crystalline gold nanowires using orange peel extract [22].

All these microbially synthesized metallic nanostructures clearly outperform chemical and physical processes by providing a better eco-balance, being faster and capable of being producing in larger scales [23]. However, a big issue of bioproduction is still the control of NPs with a certain size and shape [24]. Nevertheless, studies to analyze data on the diversity of shapes and sizes of NPs obtained by green synthesis are scare [25,26]. There are numerous variables, such as pH, metal ion concentration in the solution, adsorbent dose, contact time, etc., that affect microbial NP bioproduction [27]. Therefore, it is challenging to find the crucial points for the generation of specific NPs and optimize relevant parameters to examine the best practice [28].

Within our group, we already demonstrated the capability of biosorption and accumulation of precious and RE elements, such as Au, Eu and Sm, leading to well-defined nanosized particles [15,16,29,30]. Cyanobacteria of the genus *Anabaena* sp. are a species well suited for the intracellular uptake of precious metals, such as Au, Ag, transition metals (e.g., Cu, Al, etc.) and various REs [31,32].

Furthermore, they are good candidates to replace synthetic routes, which often require toxic solvents or produce undesirable waste products. They are easy to handle in contrast to some other microorganisms, why they have attracted increasing attention among scientists worldwide [33]. The kinetics and mechanisms of NP formation need to be understood before extending laboratory experiments to higher capacity processes [28]. If production could be accomplished by natural bioorganisms in effluents from treatment plants, this would be very beneficial. On the one hand, the algae could serve to minimize metal toxicity in the aqueous environment, and on the other hand, elemental metal would be recovered from the ions by biosorption through regular harvesting [34].

In this study, the influence of the contact time of *Anabaena* sp. with the metal-containing nutrient solution on particle sizes and shaping was investigated. High-resolution transmission electron microscopy images showing Au- and Eu-NPs formed in *Anabaena* sp. were analyzed using digital image processing. It was found that the particle growth time factor can affect particle size and number, but has less effect on particle shaping.

## 2. Materials and Methods

Biosorption experiments were performed according to our previous work [15,16]. Stock culture of *Anabaena* sp. (SAG 12.82, Algae Culture Collection (SAG) Göttingen, Germany) was transferred in a sterile 250 mL Erlenmeyer flask filled with 150 mL modified Bold’s Basal Medium (BBM, pH 6.8). All biomass suspensions were kept at a low nitrate concentration of 50% in order to increase heterocyst growth. Incubation took place at 22 °C and pH 7.3 with a 12 h day–night cycle simulation (4200 K) under continuous mixing by an orbital shaker in a temperature-controlled incubator. Appropriate samples were separated in half, one was taken as a reference and the other was incubated with the respective salt BBM solutions of HAuCl_4_ or Eu(NO_3_)∙6H_2_O, each 1 × 10^−4^ mol/L (ABCR GmbH, Karlsruhe, Germany). Aliquots of 2 mL of the biomass suspensions incubated with Au were taken after 24 h and 51 h, and of the samples mixed with Eu after 10 h and 244 h. Centrifugation (14,000 rpm, 15 min, 16,000× *g*) was used to extract biomass from the medium and the biomass pellet was prepared for TEM measurements. The supernatant was used for inductively coupled plasma mass spectrometry (ICP-MS) to prove the uptake of metal ions. More details can be taken from [15,16,30].

Prior to TEM-imaging, samples were fixed with glutaraldehyde and osmium tetroxide, dehydrated and embedded in epoxy resin, according to standard procedures. Ultrathin films (~60 nm) were cut with an Ultracut EM UC6 ultramicrotome (Leica Microsystems, Wetzlar, Germany) using a diamond knife (type ultra 35°, Diatome, Biel, Switzerland). Films were placed on pioloform-coated copper grids (Plano, Wetzlar, Germany), stained with uranyl acetate and lead citrate. The method follows established procedures [35] and has previously been described in more detail [15,16]. Nanoscale imaging was performed on a HT-7700 TEM 7700 (Hitachi, Tokyo, Japan) in high-resolution (HR) imaging mode. The system was operated at an accelerating voltage of 100 kV.

For digital image processing, *ImageJ* 1.53d (National Institute of Health, Rockville, MD, USA, freeware) with Java^TM^ version 1.8.0_112 (64-bit) under Windows 10 Pro edition was used to identify all pixels that contribute to recorded Au or Eu particles. A Fujitsu Siemens H19-1 monitor (resolution of 1280 × 1024, refresh rate of 60.020 Hz) was applied for the evaluation. The bit depth was 8-bit, the color format was RGB and the color space was SR (Standard Dynamic Range). The graphics card used was Intel(R) HD Graphics 620. TEM images were implemented as JPG (resolution 3296 × 2563 pixels, depth 24-bit), whereas the registered scale bar served as a known distance for the transfer to the particle units. To capture particles more accurately, images were cropped, smoothed and sharpened before individual thresholding took place. An appropriate size range was set for each image, to remove artefacts and cell compounds at the upper scale and scattered pixels originating from the background noise at the lower end. Particles touching the edges were excluded. Results were displayed as equivalent circular diameters (*ECD*, Equation (1)) in nm,

(1) ECD=4⋅Aπ

assuming that the particles are perfectly spherical. Since this assumption does not correspond to reality and most particles are irregular in shape by nature, the Feret major axis ratio (*FMR*, Equation (2)) and the reciprocal aspect ratio (*RAR*, Equation (3)) were calculated from the Feret diameter *D_f_* and the major *a* and minor axes *b* of the fitted ellipses for shape analysis.

(2) FMR=Dfa

(3) RAR=ba

The parameters used in Equations (1)–(3) were taken from Igathinathane’s object identification strategy and are standard outputs generated by *ImageJ* to evaluate particles shape and size [36]. The exact procedure for particle size and shape analysis can be found in detail in the previous work of the authors [37].

## 3. Results and Discussion

### 3.1. Exemplary TEM Images with Nanoparticles

**Figure 1:**
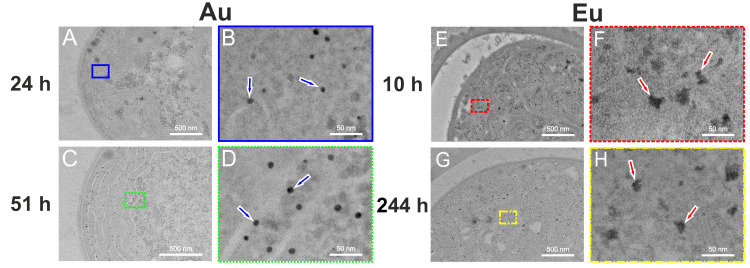
TEM images of vegetative cells of *Anabaena* sp. showing Au-NPs after 24 h (**A**,**B**) and 51 h (**C**,**D**) and its heterocysts with Eu-NPs after 10 h (**E**,**F**) and 244 h (**G**,**H**). Indicated are magnifications (**B**) (blue outline) of (**A**,**D**) (green dotted) of (**C**,**F**) (red dashed) of (**E**), and (**H**) (yellow dotted and dashed) of (**G**). Blue arrows mark some selected Au-NPs and red arrows Eu-NPs.

TEM measurements clearly show Au and Eu particles inside the cells of *Anabaena* sp. (Figure 1). After 24 and 51 h of in vivo contact time with the Au^3+^ containing solution (respectively, 10 and 244 h with the Eu^3+^ solution), cells of the living organism fully incorporated these ions, and generated nano-sized Au and Eu particles, respectively. Au-NPs were formed only in vegetative cells (Figure 1A–D), whereas Eu-NPs were generated exclusively in its heterocysts (Figure 1E–H). In high-resolution (HR) imaging, the Au-NPs (some indicated by blue arrows in Figure 1B,D) look more spherical and electron denser than the irregularly shaped Eu-NPs (red arrows in Figure 1F,H) at first glance. Therefore, the particle formation in different cells obviously affects the shape and size of the generated particles.

### 3.2. Particle Size Distributions (PSDs)

#### 3.2.1. PSDs of all NPs in Complete Cells

**Figure 2:**
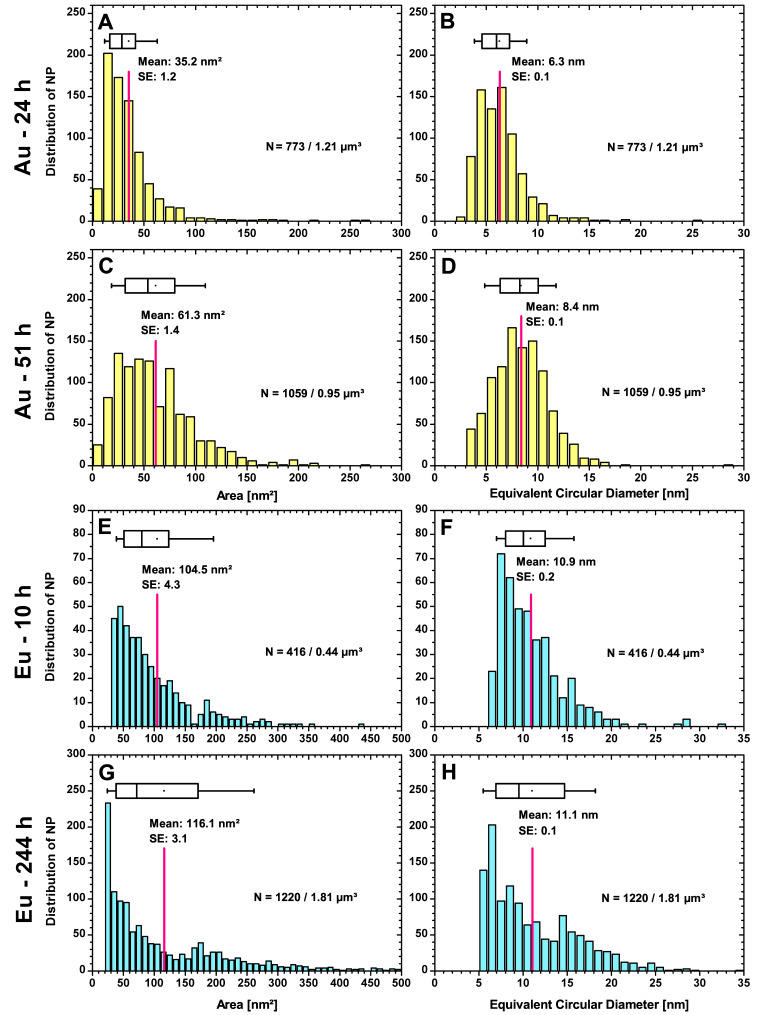
PSDs for Au-NPs after 24 h (**A**,**B**) and 51 h (**C**,**D**) and for Eu-NPs after 10 h (**E**,**F**) and 244 h (**G**,**H**) with the mean value and standard error (SE). The number of particles *N* per investigated cell volume and the average area or ECD (red lines) are indicated in the plots. The ECDs are calculated from the areas assuming round particles. The boxplots show the 25% and 75% percentiles, with the whiskers at 10% and 90%.

The PSDs of the Au- and Eu-NPs shown in Figure 2 were determined from the TEM images provided in the Supporting Information Figure S1 (Supplementary Materials Section S1 Used Datasets) and the settings listed in Table 1 during digital image analysis.

The results of Figure 2 give an indication of the average NP-forming capacity of the investigated cell volumes and their PSDs in complete cells. The vegetative cells forming Au-NPs have a total volume of 1.21 µm^3^ for cells isolated after 24 h of contact time and a volume of 0.95 µm^3^ for cells extracted after 51 h. Each cell area was measured by digital image analysis and the cell volume was calculated by assuming the sliced ultrafilms have a thickness of 60 nm. In these cell volumes, 773 particles were detected after a growth time of 24 h and 1059 Au-NPs after 51 h. For Eu, a total of 416 NPs could be registered in a cell volume of 0.44 µm^3^ (10 h) and 1220 of them in a volume of 1.81 µm^3^ (244 h). To facilitate comparison, the values are converted to a standard volume of 1.00 µm^3^. This results in the fact that with the same cell volume, about 639 particles would have formed with a growth time of 24 h and about 1115 Au-NPs with 51 h. The increase rate of particle number at slightly more than twice the time is about 57%. In the case of Eu, the situation is completely the other way round. Here, about 946 Eu-NPs would have formed at 10 h and only 674 at 244 h. It remains questionable whether this decrease of nearly 29% in the number of Eu-NPs is due to a partial agglomeration of the amorphous particles. Considering the range from approx. 300 nm^2^, it can be seen that larger particles formed after 244 h compared to after 10 h. However, there is still a large number of small particles (<50 nm^2^). The flatter distribution from about 100 nm^2^ (244 h) and from about 200 nm^2^ (10 h) indicates that, with increasing growth time, there is a higher tendency towards larger particles as well as a significantly higher number of small Eu-NPs. When analyzing images with *ImageJ*, manual adjustments must be made due to the different acquisition parameters. For example, the zoom varied for Au-NP evaluation from 7.0 k to 15.0 k, depending on the different cell sizes, which affects the selected scale and analysis range. In the analysis, it is important to place restrictions (a range), so that cellular components such as ribosomes or vacuoles with a similar or same gray scale to the Au-NPs are not included. Different measurement limits (lower/upper area limit in Table 1) result from the fact that the ranges have to be selected differently. For images No. 2 and No. 5 (not shown here, please refer to the Supplementary Materials Section S1 Used Datasets), an upward restriction was not necessary because the image section did not contain any artifacts. The limit is transferred by converting the particle size given in the area to ECD (lower/upper ECD limit in Table 1). The threshold varies slightly (0.19-0.21) due to the different brightness levels of the TEM images. The evaluations of cells No. 1-4 are summarized in Figure 2A,B and those of No. 5 and 6 in Figure 2C,D (for cell images see Supplementary Materials Section S1 Used Datasets).

Most Au-NPs (26.2%) in the size distribution (Figure 2A) belong to the size class 10–20 nm^2^, followed by the second largest number (22.4%), which have an area of 20–30 nm^2^. The average value is 35.2 nm^2^, as there are still isolated particles (≤0–4) between 90 nm^2^ and 300 nm^2^. The majority of Au-NPs (83.2%) has a size between 10 and 50 nm^2^ after 24 h, so the size range is much narrower than after 51 h (Figure 2C). Here, the average value is much higher at 61.3 nm^2^ and the distribution ranges up to 150 nm^2^ with a much higher number of particles. In contrast to 24 h, 87.3% of the particles are found in a size range of 10–100 nm^2^, which is twice as large after 51 h of growth time. To get a better impression, the areas were converted to ECD, assuming spherical particles. After 24 h (Figure 2B), the Au-NPs have an average diameter of 6.3 nm, which expands to 8.4 nm after 51 h (Figure 2D). About 20.4% of the Au-NPs have an ECD between 4 and 5 nm, which is approximately equal to the number of NPs exhibiting an ECD between 6 and 7 nm (20.8%). The intermediate class (5–6 nm) has slightly fewer Au-NP with an ECD of 17.5%. Even after 51 h, the size distribution shows a local minimum at 8–9 nm. Among the adjacent classes, 15.7% of the Au-NPs belong to the 7–8 nm size class and 13.4% fall in the 8–9 nm range. Followed by 11.2% with a diameter between 6 and 7 nm and 10.8% between 10 and 11 nm. There are no Au-NP that have an area <9 nm^2^ and only 734 of the 1059 have an area <25 nm^2^ (or <3.4 nm and only a few <5.6 nm ECD) due to the restriction made in the particle evaluation for image No. 5 and No. 6. For the 24 h case, the measurement limit was also at an area of 9 nm^2^ (or a diameter of 3.4 nm), although for some particles this limit had to be set higher to avoid counting false positives.

When looking at Figure 2F, it is noticeable that for the 416 Eu-NPs registered, the average ECD is 8.4 nm, with the dominant class here, between 8 and 10 nm, covering 44.0% of the particles. Conversely, the average ECD decreases by 1.2 nm to 7.2 nm at about a 25th growth time, and of the 1220 particles, 53.4% likely belong to the 5–10 nm (Figure 2H). This also confirms the increasing agglomeration over time.

Considering that the ECD is calculated assuming all NPs to be spherical, which is more or less the case depending on the particle shape, the unadjusted PSDs (Figure 2A,C,E,G) are also reported in area sizes for completeness, but are not discussed in detail.

#### 3.2.2. High-Resolution (HR)-PSD of Cell Sections

**Figure 3:**
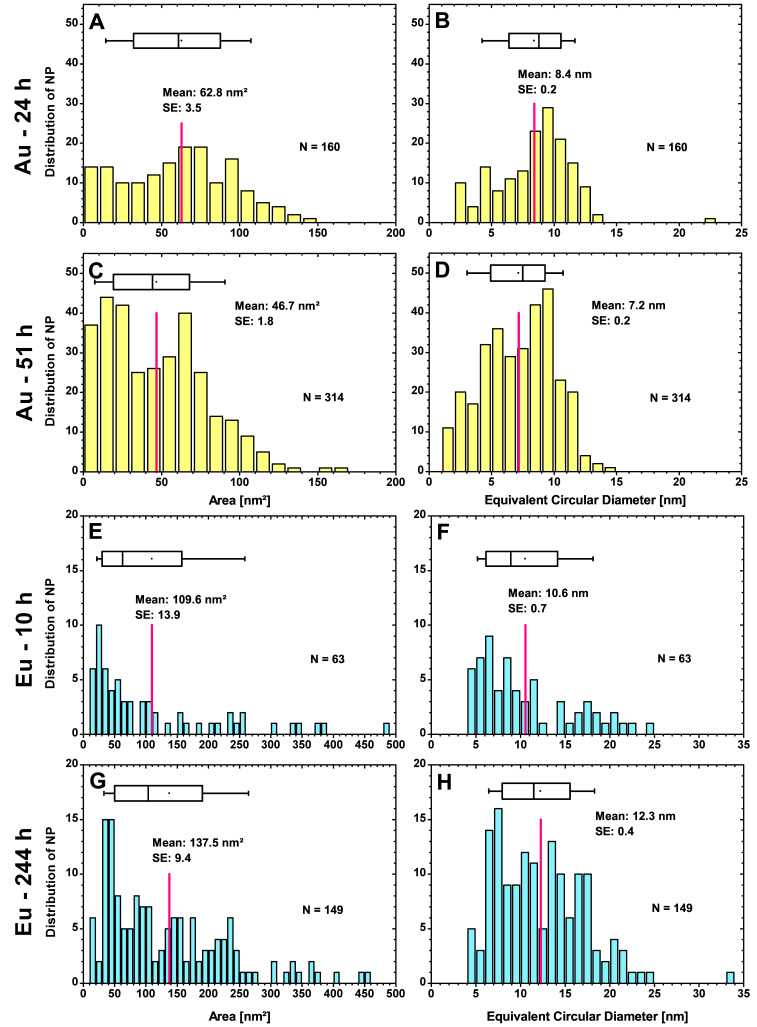
HR-PSD for Au-NPs after 24 h (**A**,**B**) and 51 h (**C**,**D**) and for Eu-NPs after 10 h (**E**,**F**) and 244 h (**G**,**H**) with the mean value and standard error (SE). The number of particles N counted in total for the same cell volume and the average area or particle diameter (red lines) are indicated in the plots. The ECDs are calculated from the areas assuming round particles. Again, the boxplots show the 25% and 75% percentiles, with the whiskers at 10% and 90%.

The high-resolution (HR) images (No. 11–75) (Supplementary Materials Figures S2–S5) can only capture a portion of the total particle count (Au-NPs: ~20.7% for 24 h, ~29.7% for 51 h; Eu-NPs: ~15.1% for 10 h, ~12.2% for 244 h), but provide a more valuable analysis of particle size and shape. The results of the HR evaluation are presented in Figure 3 (see Supplementary Materials Table S1 for TEM image settings data for Au HR-PSDs in Figures S2 and S3, and Eu HR-PSDs in Figures S4 and S5). With the more precise size distribution (Figure 3A), the average area of the Au-NPs is larger, rises to 62.8 nm^2^ instead of 35.2 nm^2^ (Figure 2A) and the corresponding ECD increases from 6.3 nm (Figure 2B) to 8.4 nm (Figure 3B). The size classes are highest in the 60–70 nm^2^ (11.9%), 70–80 nm^2^ (11.9%), and 90–100 nm^2^ (10.0%) range. The smaller particles with sizes between 0 and 20 nm^2^ (17.5% of all particles) outweigh those between 20 and 50 nm^2^ (6.3% for 20–30 nm^2^, 6.3% for 30–40 nm^2^ and 7.5% for 40–50 nm^2^). The transfer to the ECD (Figure 3B) shows it more clearly: about half of all detected particles (45.6%) have a diameter of 8–11 nm. If the range is extended to 7–12 nm, this is already 63.1% of all particles. The count at 22–23 nm was caused by two closely spaced particles, which were scored as one particle. After a growth time of 51 h, the particles have an average area of 46.7 nm^2^ (Figure 3C) instead of 61.3 nm^2^ (Figure 2C) as in the complete cells.

Here, a relatively large number of Au-NPs have a small area of 0–30 nm^2^. After 51 h, mainly smaller particles are detected, except for the class at 60–70 nm^2^ with 12.7% total particle amount. There are no particles with an area larger than 170 nm^2^ (or ECD of 15 nm). Compared to the shorter growth time, 51.0% more particles were registered for the same number of evaluated TEM images and, thus, approximately the same cell area. Again, there is a local minimum at an area of 30–60 nm^2^, but this is higher than the areas from 80 nm^2^, which is more pronounced here compared to 24 h particle growth time. The corresponding diameters (Figure 3D) show more clearly that most particles in the two fields are between 4 and 6 nm (21.7%) and 8 and 10 nm (28.0%). Including the intermediate classes, 68.8% of all particles belong to the size range 4–10 nm. 

In the case of Eu, Figure 3F reveals that for the HR evaluation, most Eu-NPs are in the dominant classes between 5 and 7 nm with 25.4% of the detected 63 particles. The average ECD is calculated at 10.6 nm, which is quite higher than the Eu-NPs belonging to the predominant class. The reason for this is the high number of individual particles in the upper size classes, shifting the mean to higher values. In contrast to the Au-NPs with ECDs ranging up to 15 nm, the Eu-NPs reach ECDs of up to 25 nm, and for 244 h, even a particle with an ECD of 34 nm is recorded. This much larger particle is the result of a fusion of two particles. For the 149 detected Eu-NPs with a contact time of 244 h, the average ECD is 12.3 nm. Thus, there is a clear growth in particle size with time, of which most particles are found in the 6–8 nm, 10–12 nm, and 13–15 nm classes. In contrast to the PSDs of the complete cells, the average ECD decreased only minimally by 0.3 nm for a contact time of 10 h and increased by 1.2 nm for 244 h. However, by the much more accurate HR analysis, only 15.1% (10 h) and 12.2% (244 h) of all Eu-NPs within the cell could be covered. The discrepancy can be explained by the missing particles and the greater susceptibility to error due to the small magnification scale.

#### 3.2.3. Local PSDs within an Exemplary Cell

**Figure 4:**
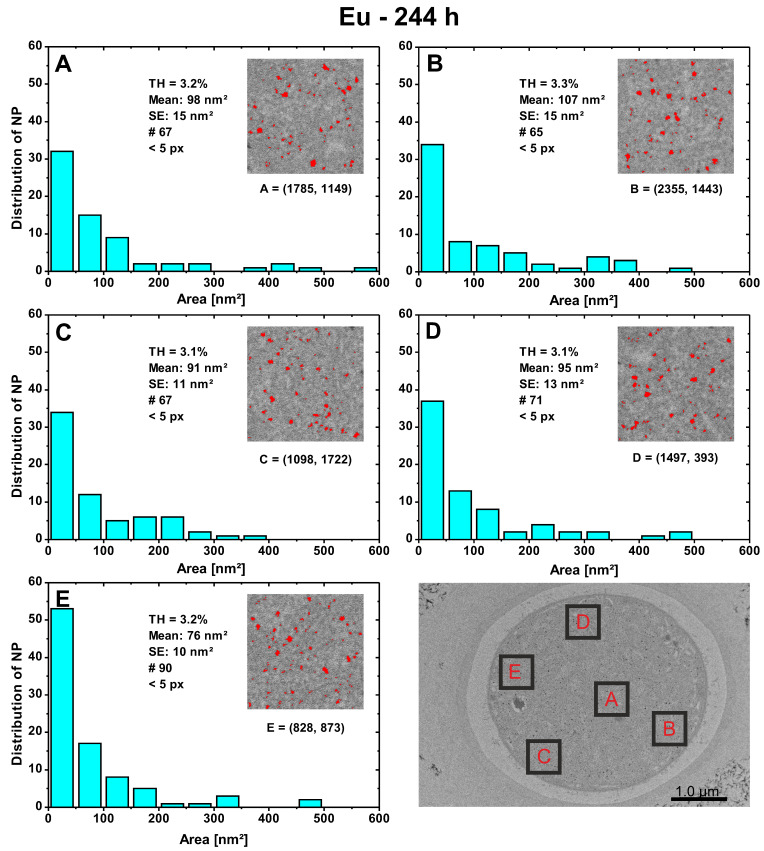
Local PSDs for Eu-NPs after 244 h growth time of five randomly selected 500 × 500 nm^2^ areas (**A**–**E**) within the presented heterocyst No. 10. Indicated in the plots are the thresholds (TH), the mean values with the standard error of the mean (SE), the total number of particles detected (#), the lower measurement limit in pixels (px), and the local (x,y)-coordinates for each digital image analysis.

To determine if the PSDs differed locally within the cells, five sites were randomly selected and local PSDs (Figure 4 and Supplementary Materials Figures S6–S8) were performed for them.

These should provide information on whether the particle number or size is uniformly distributed within the cells. All tests indicate that there are no local hotspots for either Au or Eu where NPs are formed more frequently or where particles are larger/smaller than elsewhere. Results for Au are summarized in the Supplementary Materials Section S2 (Supplementary Materials Figures S7 and S8) for the incubation time of 24 h and 51 h, as well as for Eu at 10 h. As an example, the local PSDs for the detected Eu-NPs with a contact time of 244 h (Figure 4) are discussed here. Since the locations within the cell have similar gray levels, the threshold values (TH) vary only slightly. It is important to set a lower limit for the particle size (<5 pixels), otherwise even single pixels of the adjusted grayscale value will be counted as particles. A maximum limit is not necessary if there are no artifacts. In case of artifacts, an upper value can be selected as a limit. In area E, the particle count of 90 is slightly higher than the average of ~67 for the other zones. The particle areas are largest for the classes 0–50 nm^2^, with area E having 58.9% of all particles in this class. This is slightly higher than the other areas (A: 47.8%, B: 52.3%, C: 50.7%, D: 52.1%) with an average value of only 50.7%. Most of the largest particle areas are located in area A inside the cell, followed by area D, which is close to the cell membrane. Approximately equal numbers of particles in the different classes with an area size of 50 nm^2^ and larger are found in area B. However, the differences between the areas are not meaningful enough to identify local hotspots or agglomeration. Similarly, no significant changes were observed in Eu-NPs among the five different zones at 10 h contact time (Supplementary Materials Figure S6). There is also no apparent preferential accumulation of Au-NPs during the 24 h or 51 h growth period at cellular constituents or local sites, indicating a greater particle number or size (Supplementary Materials Figures S7 and S8).

### 3.3. Shape Classification

**Figure 5:**
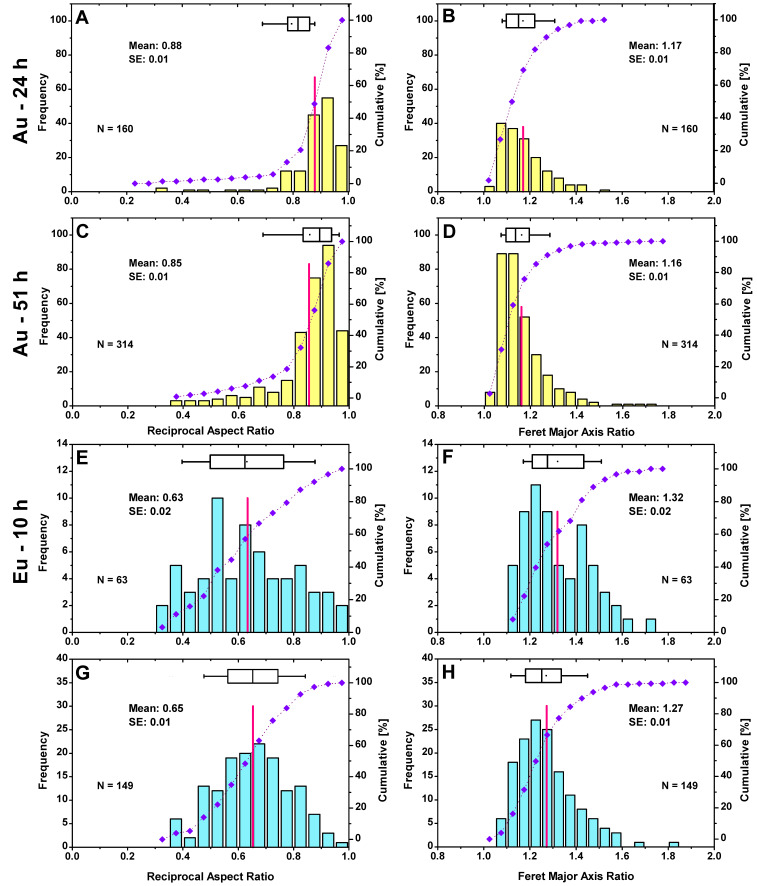
Shape classification for Au-NPs after 24 h (**A**,**B**) and 51 h (**C**,**D**) and for Eu after 10 h (**E**,**F**) and 244 h (**G**,**H**) by using RAR and FMR as shape parameters (see Equations (2) and (3)). The total particle number N, mean values (red lines) with standard errors (SE), and the cumulative frequency in percentage (purple curves) are indicated in the plots. The boxplots show the 25% and 75% percentiles, with the whiskers at 10% and 90%.

Figure 5 reveals that the Au-NPs have an average RAR (red lines) of 0.88 for 24 h and 0.85 for 51 h, with most of the particles (%) being in the 0.85–0.95 class (Figure 5A,C). Additionally, the FMR with an average value of 1.17 and 1.16, respectively, indicates that the parameters are approximately the same for both times and the main weighting falls on the classes between 1.05 and 1.2 (Figure 5B,D). In the case of Eu, the particle shapes exhibit a more diversified spectrum (0.3–1.0) than those of Au-NPs, where the dominant classes are mainly found in the range of 0.7–1.0. The RAR is 0.63 for the detected 63 particles after an incubation time of 10 h and 0.65 for the 149 particles after 244 h. The small difference in the mean values at the different times suggests that the particle shapes are only imperceptibly affected by the change in growth time. A comparison of the dominant classes also does not reveal a strongly differentiated pattern. For the FMR, the distribution is dominant both times for the classes at 1.2–1.3. Again, a frequent particle shape is observed, with greater variance than for Au.

To obtain a more descriptive shape classification, a subdivision into the shape classes “very angular” to “very round” was made according to the RAR interval assignments shown in Table 2.

**Figure 6:**
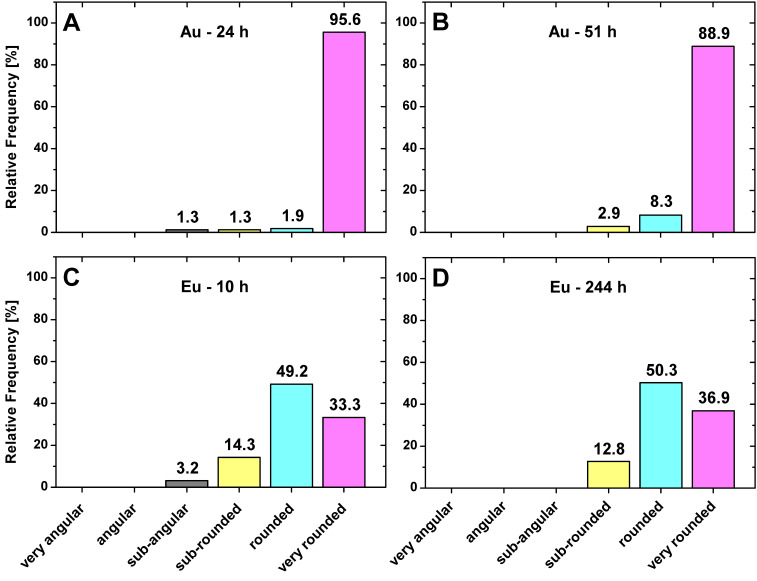
Shape classification into six different classes for Au-NPs after 24 h (**A**) and 51 h (**B**) growth time and for Eu-NPs after 10 h (**C**) and 244 h (**D**) with the indication of percentages.

It can be seen that the majority of all Au-NPs are very rounded, 95.6% for 24 h and 88.9% for 51 h (Figure 6A,B). Increasing the time factor leads to an absence of sub-angular Au-NPs, but in return, less are very rounded. In the case of Eu-NPs, about 50% of the particles are just rounded for both incubation times. The remaining particles are mainly classified to very rounded (33.3% for 10 h and 36.9% for 244 h), followed by sub-rounded (14.3% for 10 h and 12.8% for 244 h). These results show that the Eu-NPs are round enough to justify the previous calculations of the ECD assuming spherical particles. For the shorter growth time (10 h), some Eu-NPs are also found in the sub-angular class, which disappear with increasing time. It appears that the initially more irregular amorphous Eu-NPs agglomerate into rounder particles over time. On the one hand, with increasing growth time, some of the crystalline Au-NPs become a little more irregular. On the other hand, there are less angular exotics. However, the changes are so minor that varying the incubation time does not significantly affect the overall shape of the particles.

Previous studies have shown that microbial synthesis and growth of metal NPs depend on the initial concentration of the respective metal ions, in addition to other factors such as adsorbent dose, pH value, temperature, and contact time. At the same time, this also affects the size, shape and agglomeration of the NPs [38,39,40]. In similar studies [19,41], higher initial concentrations of HAuCl_4_ (0.5 and 1.0 mM, and 25 mg/L resp. 0.13 mM) at incubation times from 24 h up to several days resulted in more diverse shapes, e.g., spherical, triangular, hexagonal and irregular ones, with even larger NP sizes (30 nm up to 100 nm). However, in the case for *Anabaena laxa*, the higher concentrations of 0.5 and 1.0 mM also led to the death of the cells used within the 24 h [19]. Rösken et al. made a similar observation for *Anabaena* sp. with initial concentrations of 0.8 mM HAuCl_4_, where cell viability ceased within a week [16]. In contrast, at lower concentrations of around 0.1 mM and slightly higher, there appears to be a preference for smaller and more spherical gold NPs [19,41].

In this biorecovery process, metal ions are adsorbed via the cell surface and subsequently reductively converted to NPs in the presence of enzymes synthesized by the microbes. The transformation occurs both intracellularly and extracellularly, and the metal nanoparticles are deposited accordingly, either inside or around the cell [38]. Studies by Chakraborty et al. [42] showed that *Cyanophyceae*, *Lyngbya majuscule* and *Spirulina subsalsa* actively absorb Au ions within 72 h of exposure, with absorption maxima greater than 96% and 86%, respectively. After processing the washing agent ethylenediaminetetraacetic acid, which chelates metal ions absorbed at the exterior of the cell, the intracellular fractions are obtained after appropriate workup. It turned out that for both organisms, gold in 20% amount for *Lyngbya majuscule* and 50% for *Spirulina subsalsa* could be determined at the cell surface, thus decreasing the intracellular yield and consequently the NPs production. 

Therefore, the here used *Anabaena* sp. appears to effectively produce stable gold NPs intracellularly without agglomeration within a shorter time (Au-NPs within 24 h and for Eu within 10 h), but this would need to be verified by a more comparative study. For Eu-NPs, comparisons are not very meaningful since data in this field are still very sparse [43].

## 4. Conclusions

In this study, the influence of incubation time on the bioproduction of metallic gold (Au) and amorphous europium (Eu) nanoparticles (NP) in the cyanobacterium *Anabaena* sp. was investigated. Digital image processing was used to analyze HR-TEM images showing that Au- and Eu-NPs formed in the cyanobacterium. Special attention was drawn to the development of the size and shape classification of the nanoscale, bioproduced particles. Increasing the incubation time from 24 h to 51 h results in an almost double Au particle number. The average equivalent circular diameter (ECD) shrinks from 8.4 to 7.2 nm, with two predominant particle size classes (4–6 and 9–10 nm), instead of previously only one (8–10 nm). For the microbial formed amorphous Eu-NPs with a significantly higher growth time factor, an increasing average ECD from 10.6 to 12.3 nm, distributed in three classes (6–8, 10–12 and 13–15 nm), is found for 244 h compared to just the single class at 5–7 nm for 10 h. The factors leading to a decrease in particle productivity of almost 29% in the number of Eu-NPs with higher growth time are still questionable and have been attributed to partial agglomeration of the amorphous particles.

A comparison between five local particle size distributions (PSDs) within one cell compartment show no hotspots of preferred agglomerate zones. 

Shape classification was performed by using the reciprocal aspect ratio (RAR) and Feret major axis ratio (FMR) as shape parameters. For the detected Au-NP, the RAR and FMR has no significant changes for the different growth times. The formed NPs are clearly classified in both cases as very rounded, 95.6% at 24 h and 88.9% at 51 h contact time. Compared to the Au-NPs, the shape of the Eu-NPs is less “rounded” and also shows no time-dependent shape effects, but has a broader shape spectrum. Therefore, half of the Eu-NP shapes are classified to be “rounded”, followed by “very rounded” (~35%) and then “sub-rounded” (~13%). Thus, a higher growth time factor only very slightly enhanced the tendency toward more spherical particles.

*Anabaena* sp. was able to bioform NPs of very small size with a high tendency toward spherical shaped NPs within a very short time. For short growth time factors, no agglomeration and a relatively high productivity could be recorded. This makes these particles interesting for many applications. The initial salt concentrations used were lower, but resulted in better efficiencies in comparable studies.

The results showed only negligible time-dependent shape effects, but more obvious size effects. Since biosynthesis is more difficult to control and a variety of factors influence particle growth, further investigation is needed to obtain tailor-made particles.

Furthermore, it was found that cyanobacteria of the genus *Anabaena* spec. can be successfully used to extract raw materials from aqueous solutions containing trivalent Au or Eu ions by recovery means. Thus, *Anabaena* sp. is an appropriate and potential candidate for a cost-effective and non-toxic synthesis of the specific NP sizes and shapes presented in this work by just varying the growth time. A scale-up procedure is potentially feasible. Since crystalline Au-NPs are formed only in its vegetative cells and Eu-NPs only in its heterocysts, a separation process would be conceivable.

## Figures and Tables

**Table 1:** Underlying TEM image settings for the determination of Au- and Eu-NP sizes in the complete vegetative cells (Au) and heterocysts (Eu), respectively (inf = infinity).

Ion Contact Time	Cell No.	Zoom	Range [px]	Scale Ratio [px/µm]	Threshold [%]	Counts
Au—24 h	1	×8.0 k	5–500	736	0.2	178
	2	×8.0 k	10–inf	732	0.2	30
	3	×15.0 k	10–inf	1374	0.2	194
	4	×10.0 k	10–300	917	0.2	371
Au—51 h	5	×8.0 k	5–inf	734	0.4	552
	6	×7.0 k	10–1000	639	0.5	507
Eu—10 h	7	×7.0 k	15–500	643	1.4	416
Eu—244 h	8	×5.0 k	5–100	458	0.5	742
	9	×7.0 k	20–400	639	1.3	258
	10	×6.0 k	40–300	496	1.4	220

**Table 2:** Interval assignments of the RAR values into six shape classes.

Class	Very Angular	Angular	Sub- Angular	Sub-Rounded	Rounded	Very Rounded
RAR value	0.12–0.17	0.17–0.25	0.25–0.35	0.35–0.49	0.49–0.70	0.70–1.00

## Data Availability

The raw data and processed data required to reproduce these findings are available on request of the authors.

## Supplementary Materials

The following supporting information can be downloaded at: https://www.mdpi.com/article/10.3390/nano13010130/s1, Section S1 Used Datasets: Figure S1 Vegetative cells of *Anabaena* sp. showing Au-NPs and respective heterocysts with Eu-NPs; Figure S2 HR-TEM images of Au-NPs after 24 h; Figure S3 HR-TEM images of Au-NPs after 54 h; Figure S4 HR-TEM images of Eu-NPs after 10 h; Figure S5 HR-TEM images of Eu-NPs after 244 h; Table S1 TEM image settings for the analysis of Au and Eu-NPs sizes; Section S2 Local particle size distributions (PSDs): Figure S6 Local PSDs for Eu-NPs of contact time 10 h; Figure S7 Local PSDs for Au-NPs of contact time 24 h; Figure S8 Local PSDs for Au-NPs of contact time 51 h.
