# Supplementary material for: Time-Dependent Size and Shape Evolution of Gold and Europium Nanoparticles from a Bioproducing Microorganism, a Cyanobacterium: A Digitally Supported High-Resolution Image Analysis
Source: Nanomaterials (Basel). 2022 Dec 27;13(1):130. doi: 10.3390/nano13010130 (PMC9824745; doi:10.3390/nano13010130)
Supplement: Supplementary file 1 [file nanomaterials-13-00130-s001.zip › nanomaterials-2109305-supplementary.pdf]

Time-Dependent Size and Shape Evolution of Gold and Europium Nanoparticles from a Bioproducing Microorganism, a Cyanobacterium: A Digitally Supported High-Resolution Image Analysis

Melanie Fritz [1], Susanne Körsten [1], Xiaochen Chen [2], Guifang Yang [2], Yuancai Lv [2], Minghua Liu [2], Stefan Wehner [1] and Christian B. Fischer [1,3,*]

[1] Department of Physics, University Koblenz-Landau, Universitätsstraße 1, D-56070 Koblenz, Germany. melli.3882@gmail.com (M.F.); susanne.koersten@gmail.com (S.K.); wehner@uni-koblenz.de (S.W.)

[2] Fujian Provincial Engineering Research Center of Rural Waste Recycling Technology, College of Environment & Resources, Fuzhou University, Fuzhou, 350116, China. chenxiaochen@fzu.edu.cn (X.C.); abc396550322@163.com (G.Y.); yclv@fzu.edu.cn (Y.L.); mhliu2000@fzu.edu.cn (M.L.)

[3] Materials Science, Energy and Nano-engineering Department, Mohammed VI Polytechnic University, 43150 Ben Guerir, Morocco

* Correspondence: C.B.F.; chrbfischer@uni-koblenz.de; Tel.: +492612872345

Content

S.1 Used Datasets **page S – 2**

Fig. S1 Vegetative cells of *Anabaena* sp. showing Au-NPs and respective heterocysts with Eu-NPs **page S – 2**

Fig. S2 HR-TEM images of Au-NPs after 24 h **page S – 3**

Fig. S3 HR-TEM images of Au-NPs after 54 h **page S – 4**

Fig. S4 HR-TEM images of Eu-NPs after 10 h **page S – 5**

Fig. S5 HR-TEM images of Eu-NPs after 244 h **page S – 6**

Tab. S1 TEM image settings for the analysis of Au and Eu-NPs sizes **page S – 7**

S.2. Local Particle Size Distributions (PSDs) **page S – 9**

Fig. S6 Local PSDs for Eu-NPs of contact time 10 h **page S – 9**

Fig. S7 Local PSDs for Au-NPs of contact time 24 h **page S – 10**

Fig. S8 Local PSDs for Au-NPs of contact time 51 h **page S – 11**

S.1 Used Datasets

The results of particle size distributions (PSDs) of gold (Au) and europium (Eu) nanoparticles (NPs) in complete cells by digital image analysis from Fig. 2 were obtained using the TEM images in Fig. S1.

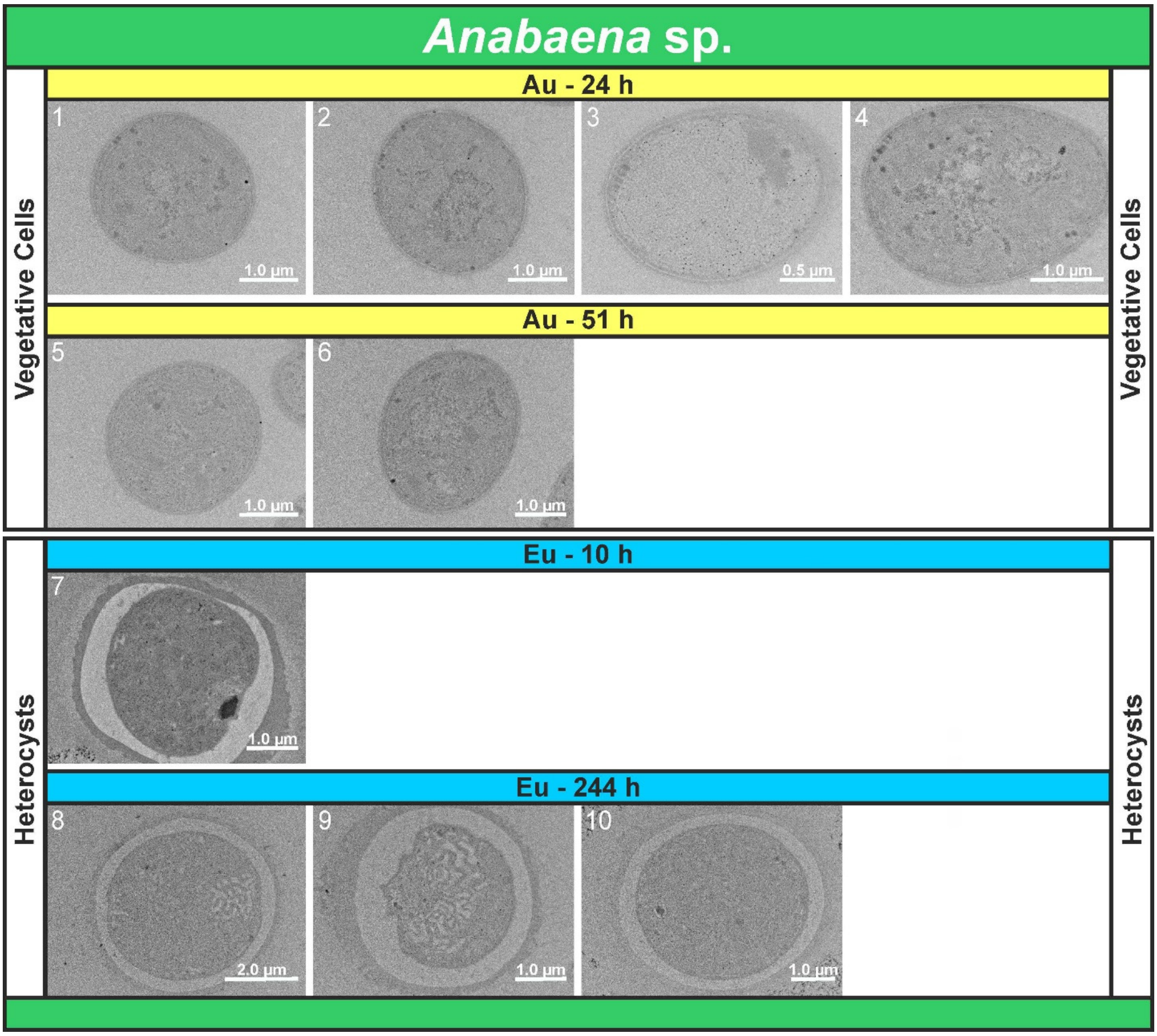

Fig. S1 Vegetative cells of *Anabaena* sp. showing Au-NPs (yellow rows) after a growth time of 24 h (No. **1-4**) and 51 h (No. **5** and **6**) and respective heterocysts with Eu-NPs (blue rows) after 10 h (No. **7**) and 244 h (No. **8-10**).

The following high-resolution (HR) transmission electron microscopy (TEM) images in Figs. S2-5 are from magnifications of these cells (compare Fig. 1 A-H). The results of particle size distributions (PSDs) in Fig. 3 were determined using the HR-TEM images in Fig. S2-5.

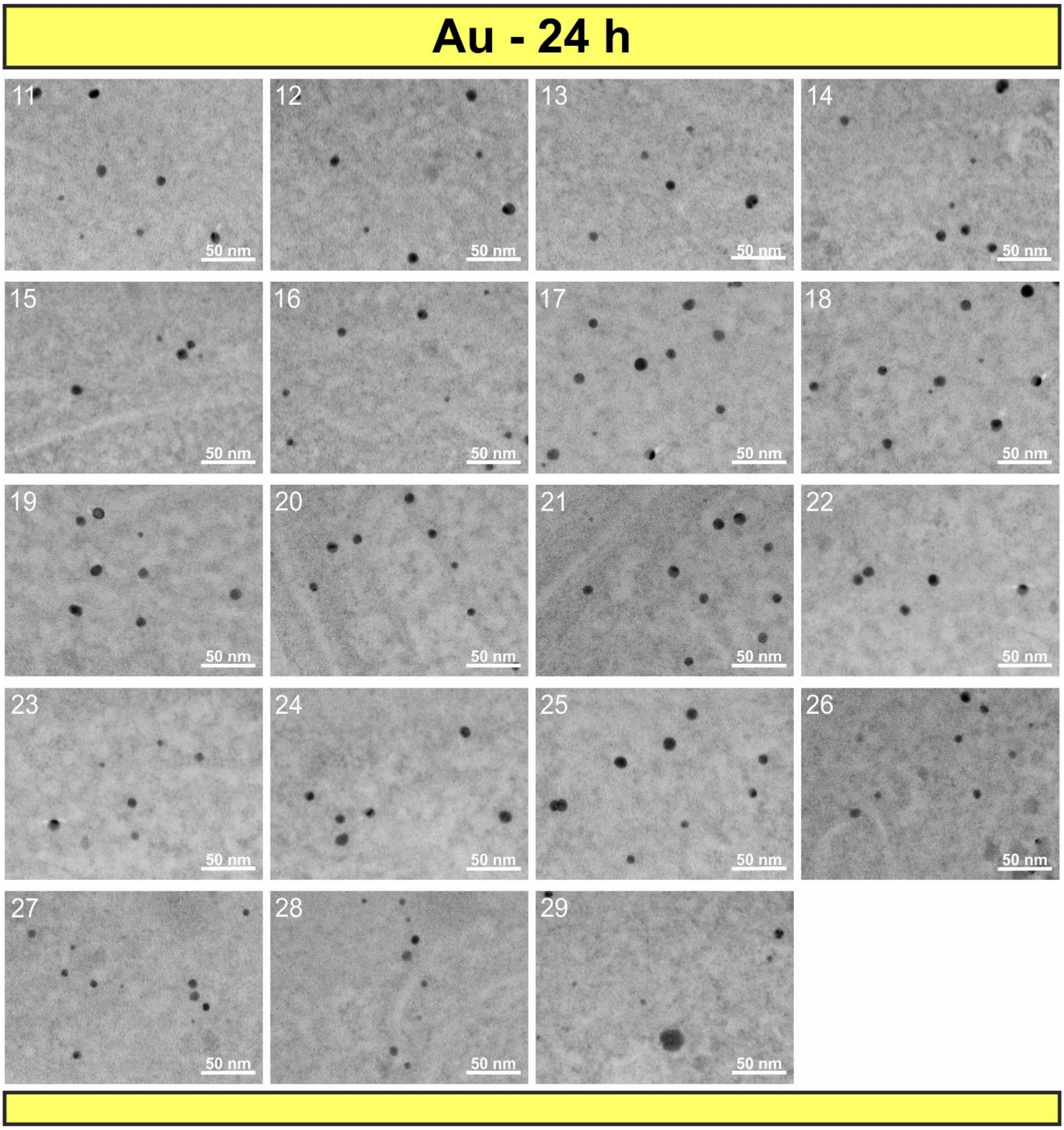

Fig. S2 HR-TEM images (No. **11-29**) show Au-NPs after a growth period of 24 h in vegetative cells of *Anabaena* sp.

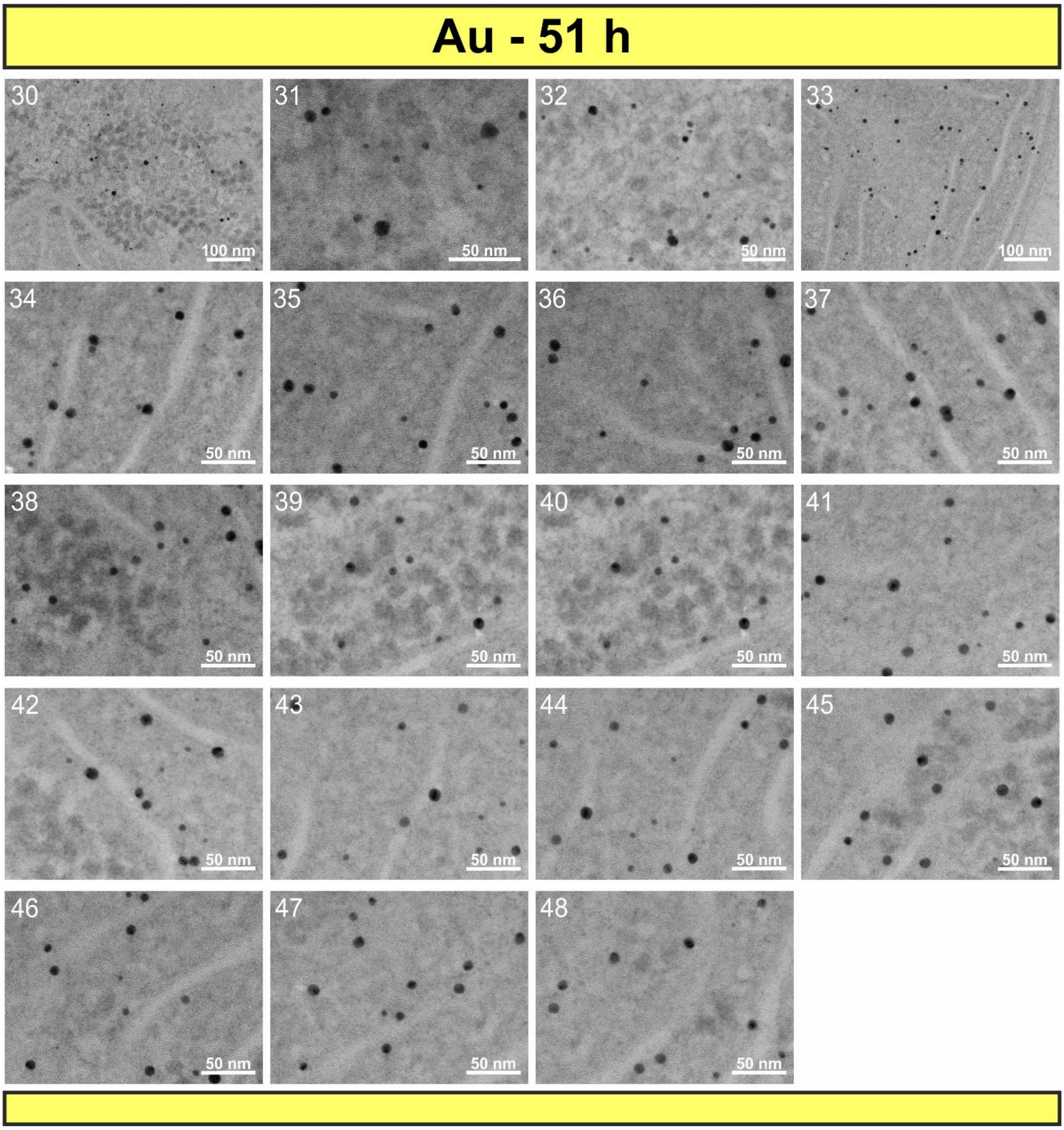

Fig. S3 HR-TEM images (No. **30-48**) show Au-NPs after a growth period of 51 h in vegetative cells of *Anabaena* sp.

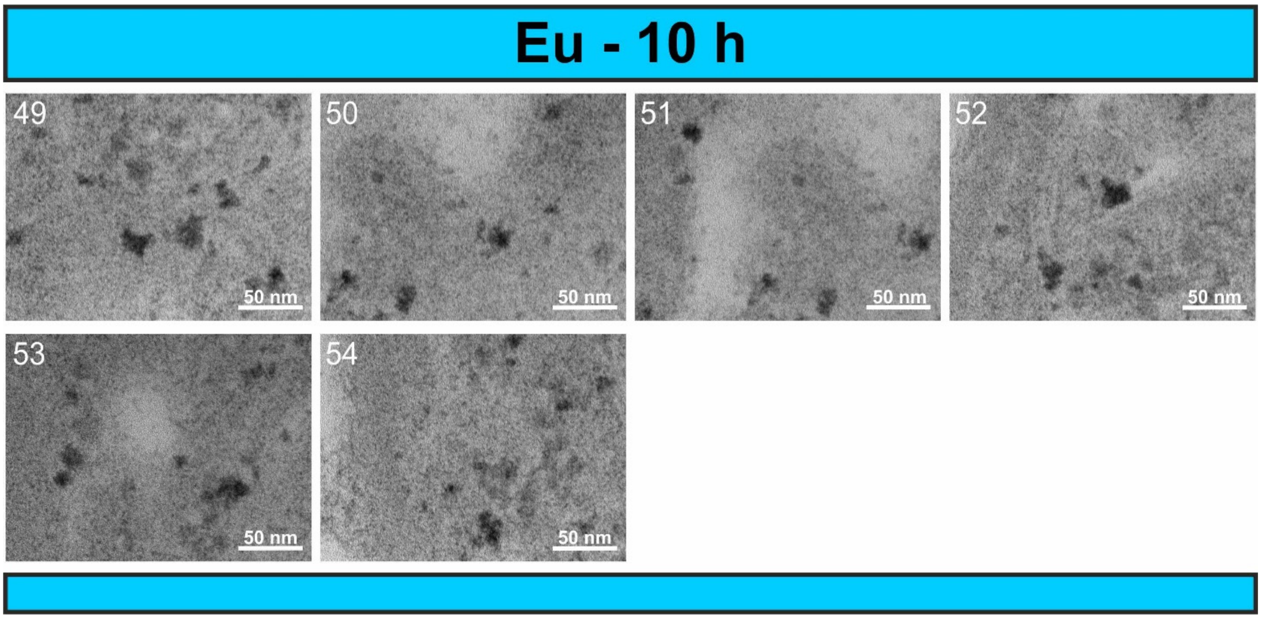

Fig. S4 HR-TEM images (No. **49-51**) show Eu-NPs after a contact time of 10 h in heterocysts of *Anabaena* sp.

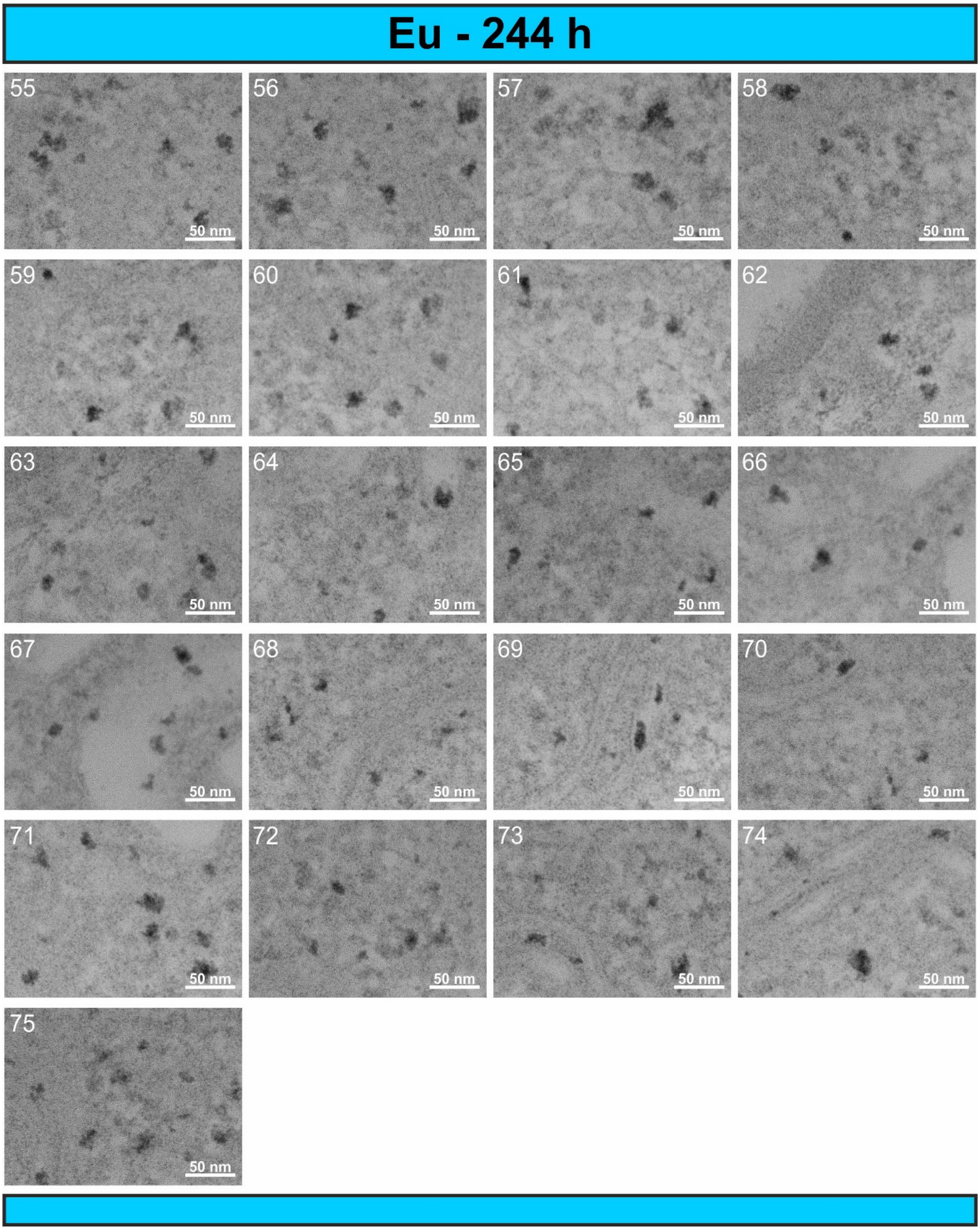

Fig. S5 HR-TEM images (No. **55-75**) show Eu-NPs after a contact time of 244 h in heterocysts of *Anabaena* sp.

The settings for the digital image analysis of Au and Eu-NP sizes in HR with *ImageJ* are listed in Tab. S1.

Tab. S1 TEM image settings for the analysis of Au and Eu-NPs sizes in HR with *ImageJ*. In addition to the counts for the respective cell No., the following parameters are displayed: Zoom factor, measurement range in px, scale ratio in px/nm, and threshold in % (inf = infinity).

Contact Time	Cell No.	Zoom	Range [px]	Scale ratio [px/nm]	Thresh old [%]	Counts
**Au 24 h**	11	x150.0k	200-inf	13.76	1.8	8
	12	x150.0k	200-inf	13.73	1.1	7
	13	x150.0k	500-inf	13.76	3.8	7
	14	x150.0k	500-inf	13.71	2.0	8
	15	x150.0k	200-inf	13.74	1.6	5
	16	x150.0k	500-inf	13.78	1.9	8
	17	x150.0k	3000-inf	13.74	11.2	10
	18	x150.0k	2000-inf	13.76	5.5	11
	19	x150.0k	1000-inf	13.68	4.7	8
	20	x150.0k	1000-inf	13.78	2.7	8
	21	x150.0k	1000-inf	13.72	3.8	11
	22	x150.0k	1000-inf	13.72	3.0	5
	23	x150.0k	1000-inf	13.74	2.3	6
	24	x150.0k	2000-inf	13.78	3.0	6
	25	x150.0k	1000-30000	13.78	3.1	7
	26	x150.0k	1500-inf	13.76	2.2	8
	27	x150.0k	1100-inf	13.76	3.6	13
	28	x150.0k	2000-inf	13.70	5.1	8
	29	x150.0k	2500-inf	13.71	6.1	6
**Au 51 h**	30	x60.0k	80-inf	5.50	0.4	39
	31	x200.0k	5000-inf	18.32	9.6	9
	32	x120.0k	500-inf	11.00	1.5	16
	33	x60.0k	50-inf	5.49	1.1	65
	34	x150.0k	100-inf	13.80	1.6	14
	35	x150.0k	250-inf	13.76	3.1	15
	36	x150.0k	250-inf	13.80	3.4	14
	37	x150.0k	500-inf	13.70	3.6	15
	38	x150.0k	3000-inf	13.76	6.9	21
	39	x150.0k	500-inf	13.72	2.1	9
	40	x150.0k	1500-inf	13.78	2.1	8
	41	x150.0k	1000-inf	13.74	2.6	10
	42	x150.0k	1000-25000	13.76	3.3	10
	43	x150.0k	1000-inf	13.76	4.7	12
	44	x150.0k	1000-inf	13.76	2.6	13
	45	x150.0k	1000-inf	13.72	2.6	11
	46	x150.0k	1000-inf	13.78	5.1	11
	47	x150.0k	1000-inf	13.78	4.6	12
	48	x150.0k	1000-inf	13.80	2.2	10

**Eu 10 h**	49	x150.0k	3000-inf	13.80	11.6	16
	50	x150.0k	8000-inf	13.74	8.9	6
	51	x150.0k	5000-inf	13.76	6.8	8
	52	x150.0k	4000-inf	13.78	8.8	10
	53	x150.0k	3000-inf	13.76	12.6	9
	54	x150.0k	3000-inf	13.74	8.3	14
**Eu 244 h**	55	x150.0k	2000-inf	13.76	13.5	6
	56	x150.0k	5000-inf	13.76	13.6	10
	57	x150.0k	10000-inf	13.76	13.6	8
	58	x150.0k	5000-inf	13.75	1.410.0	7
	59	x150.0k	20000-inf	13.72	9.6	4
	60	x150.0k	8000-inf	13.72	7.1	6
	61	x150.0k	8000-inf	13.78	6.2	4
	62	x150.0k	5000-inf	13.76	14.3	4
	63	x150.0k	10000-inf	13.76	18.6	8
	64	x150.0k	5000-inf	13.70	15.1	8
	65	x150.0k	5000-inf	13.80	9.8	7
	66	x150.0k	5000-inf	13.76	10.8	5
	67	x150.0k	5000-inf	13.78	9.2	9
	68	x150.0k	5000-inf	13.76	7.7	8
	69	x150.0k	5000-inf	13.78	5.9	5
	70	x150.0k	5000-inf	13.76	6.0	4
	71	x150.0k	5000-inf	13.74	12.0	10
	72	x150.0k	7000-inf	13.70	12.2	7
	73	x150.0k	5000-inf	13.78	8.5	7
	74	x150.0k	5000-inf	13.72	8.8	6
	75	x150.0k	3000-inf	13.78	12.0	15

S.2 Local Particle Size Distributions (PSDs)

The local PSDs for Eu-NPs at a contact time of 10 h for five randomly selected areas (A-E) within cell No. 7 (compare Fig. S1) are shown in Fig. S6. The highest number of particles (#) is 90 in B and the lowest is 68 in C. If we draw a limit at 0.1 µm² particle area, then for A: 28.6 %, B: 25.6 %, C: 29.4 %, D: 28.2 % and E: 24.3 % particles are above this, meaning the largest particles areas are located in C, followed by D. The minimal local variations in particle abundance and size are not clear enough to pinpoint a preferred region of formation or zones of enhanced accumulation.

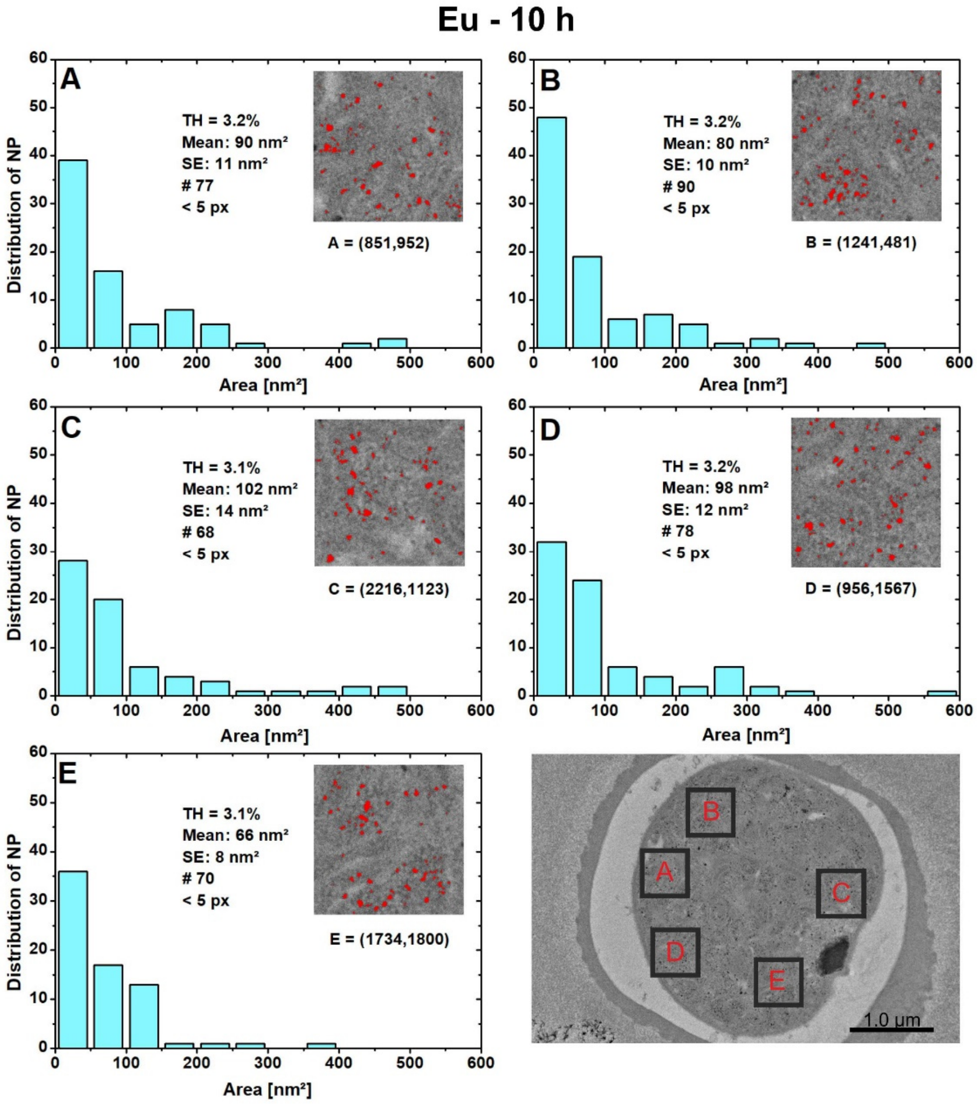

Fig. S6 Local PSDs for Eu-NPs of contact time 10 h in five arbitrarily chosen areas (**A-F**) within Cell No. 7.

The local PSDs for Au-NPs with a contact time of 24 (Fig. S7) and 51 h (Fig. S8) also do not allow clear conclusions. Local variations are indeed recognizable, e.g. for Au-NPs for 24 h growth time the particle number variance is quite large with a range between 13 and 38 (Fig. S7). However, slightly different parameters had to be selected for B and D, so the larger particle number could be due to the smaller lower limit (30 instead of 50 px). It is striking that the dominant particle areas are not found in the smallest class as in the Eu-NPs, but in the 0.05 to 0.10 range. This is particularly evident in Fig. S7D, where 78.8 % of the particles belong. Area D, along with B, has the largest contact area near the cell membrane.

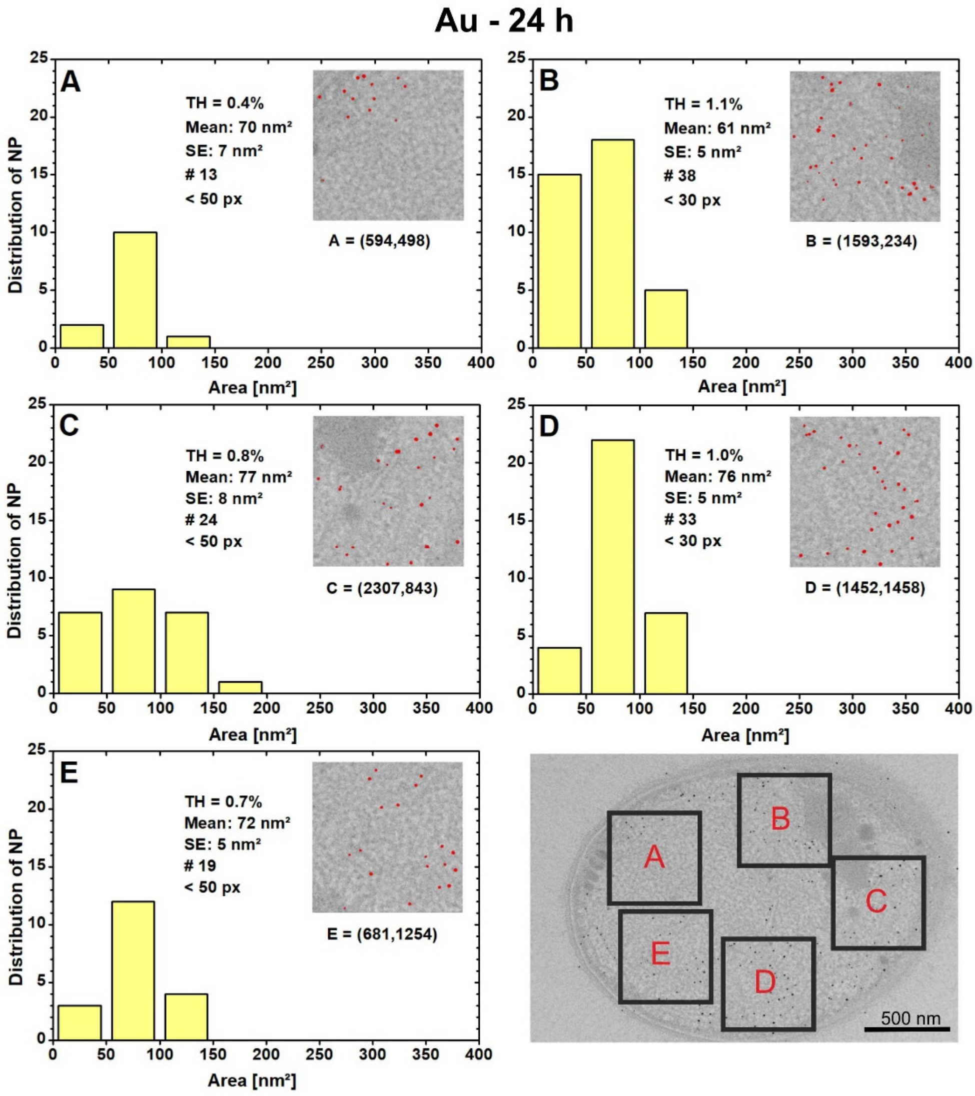

Fig. S7 Local PSDs for Au-NPs of contact time 24 h in five arbitrarily chosen areas (**A-F**) within Cell No. 3.

For 51 h growth time, as expected, more Au-NPs with larger particle areas can be identified (Fig. S8). If a limit applies at a particle area of 0.15 µm², then for A: 20.0 %, B: 17.9 %, C: 2.8 %, D: 20.0 % and E: 2.9 % of all Au-NPs are larger. Accordingly, the larger Au-NPs are found in A and D, followed by B, and the smaller particles dominate in C and E. As before, no particle agglomerations can be recognized.

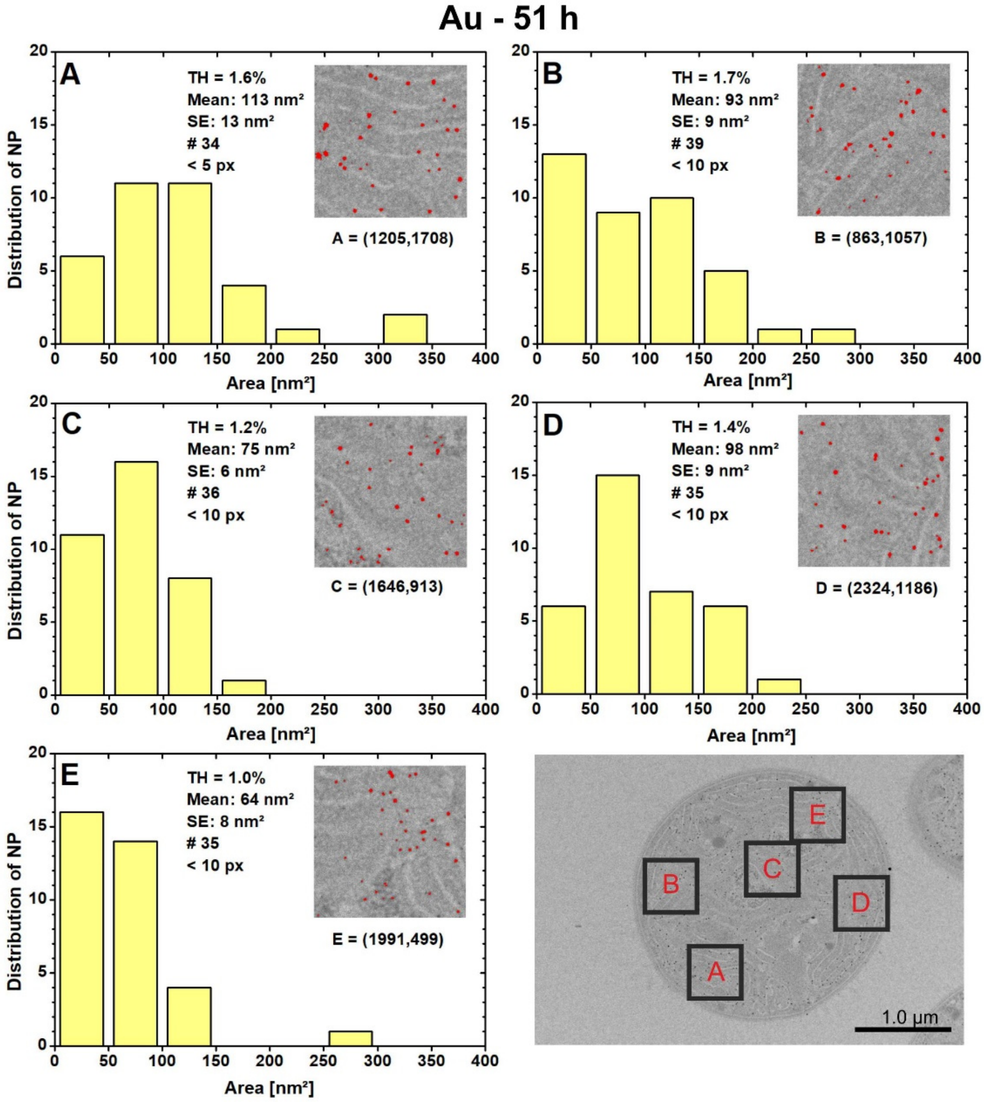

Fig. S8 Local PSDs for Au-NPs of contact time 51 h in five arbitrarily chosen areas (**A-F**) within Cell No. 5.

The threshold values vary more in the evaluations of the Au-NPs due to the structure of the vegetative cells than as in the evaluation of the Eu-NPs in the heterocysts. This difference is not avoidable, but also not significant.

The Au-NPs can be clearly distinguished from other cellular components, which is why the particle analysis can even be performed more accurately here by specific adjustment.
